# Metallic Implants Used in Lumbar Interbody Fusion

**DOI:** 10.3390/ma15103650

**Published:** 2022-05-20

**Authors:** Jakub Litak, Michał Szymoniuk, Wojciech Czyżewski, Zofia Hoffman, Joanna Litak, Leon Sakwa, Piotr Kamieniak

**Affiliations:** 1 Department of Clinical Immunology, Medical University of Lublin, Chodźki 4A, 20-093 Lublin, Poland; jakub.litak@gmail.com; 2 Department of Neurosurgery and Pediatric Neurosurgery in Lublin, Jaczewskiego 8, 20-090 Lublin, Poland; wojciech.w.czyzewski@gmail.com (W.C.); pkamieniak@poczta.onet.pl (P.K.); 3 Student Scientific Society, Medical University of Lublin, Al. Racławickie 1, 20-059 Lublin, Poland; michmatsz@gmail.com; 4 Department of Didactics and Medical Simulation, Medical University of Lublin, Chodźki 4, 20-093 Lublin, Poland; 5 St. John’s Cancer Center, Jaczewskiego 7, 20-090 Lublin, Poland; litak.joanna@gmail.com; 6 Student Scientific Society, Kazimierz Pulaski University of Technologies and Humanities in Radom, Chrobrego 27, 26-600 Radom, Poland; sakwus@gmail.com

**Keywords:** metal alloys, implants, inter body fusion, titanium, stainless steel, cobalt-chromium, nitinol

## Abstract

Over the last decade, pedicle fixation systems have evolved and modifications in spinal fusion techniques have been developed to increase fusion rates and improve clinical outcomes after lumbar interbody fusion (LIF). Regarding materials used for screw and rod manufacturing, metals, especially titanium alloys, are the most popular resources. In the case of pedicle screws, that biomaterial can be also doped with hydroxyapatite, CaP, ECM, or tantalum. Other materials used for rod fabrication include cobalt–chromium alloys and nitinol (nickel–titanium alloy). In terms of mechanical properties, the ideal implant used in LIF should have high tensile and fatigue strength, Young’s modulus similar to that of the bone, and should be 100% resistant to corrosion to avoid mechanical failures. On the other hand, a comprehensive understanding of cellular and molecular pathways is essential to identify preferable characteristics of implanted biomaterial to obtain fusion and avoid implant loosening. Implanted material elicits a biological response driven by immune cells at the site of insertion. These reactions are subdivided into innate (primary cellular response with no previous exposure) and adaptive (a specific type of reaction induced after earlier exposure to the antigen) and are responsible for wound healing, fusion, and also adverse reactions, i.e., hypersensitivity. The main purposes of this literature review are to summarize the physical and mechanical properties of metal alloys used for spinal instrumentation in LIF which include fatigue strength, Young’s modulus, and corrosion resistance. Moreover, we also focused on describing biological response after their implantation into the human body. Our review paper is mainly focused on titanium, cobalt–chromium, nickel–titanium (nitinol), and stainless steel alloys.

## 1. Introduction

Over the past few decades, lumbar spinal fusion (lumbar interbody fusion, LIF) has been recommended as a well-known, standard surgical treatment for degenerative disc disease (DDD) of the lumbar spine. DDD may cause low back pain and radicular symptoms, which can significantly decrease the quality of life. The prevalence of symptomatic DDD increases with age and occurs in 10% of the male population at the age of 50 and up to 50% at the age of 70 [1]. According to some reports, DDD may concern even 90% of the population including asymptomatic cases [2]. LIF effectively provides stabilization of painful motion segment, restores lordosis and disc height, corrects the deformity, and may provide indirect decompression of dural sac and nerve roots [3,4]. That allows immediate relief of DDD symptoms. Other indications for this procedure include traumatic injuries, degenerative or congenital deformities, spondylolisthesis, spinal stenosis, and tumors [3,5,6,7].

**Figure 1:**
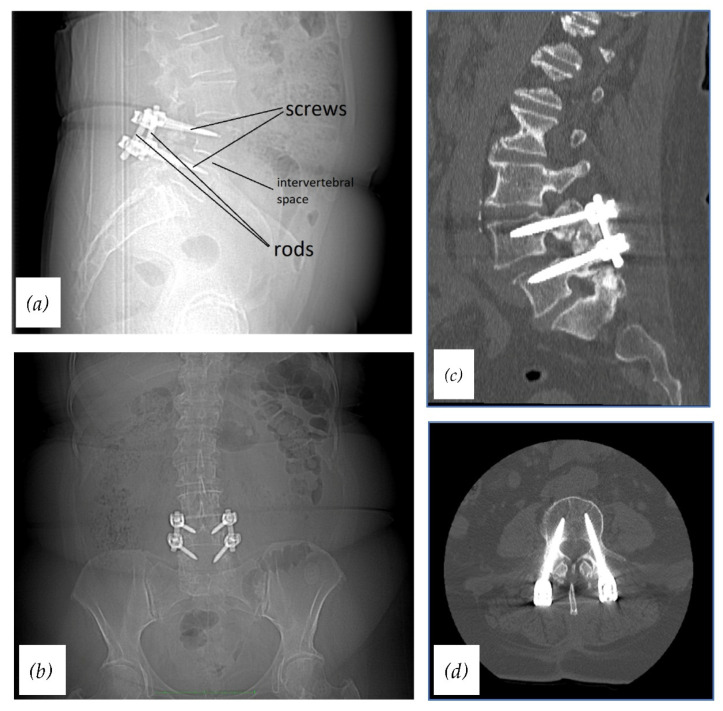
Radiographs and CTs of stabilized lumbar spine using the standard spinal fixation device: (**a**) Radiograph, lateral view (with descriptions); (**b**) radiograph, AP view; (**c**) CT, sagittal plane; (**d**) CT, axial plane.

There are various approaches to a lumbar interbody fusion. However, there is a lack of sufficient and reliable evidence to establish one of them as a standard lumbar fusion method. Posterior lumbar interbody fusion (PLIF) and anterior lumbar interbody fusion (ALIF) are the most traditional techniques. Nowadays there are other, less invasive methods including lateral lumbar interbody fusion (LLIF), extreme lateral lumbar interbody fusion (XLIF), oblique lumbar interbody fusion (OLIF), and transforaminal lumbar interbody fusion (TLIF) [6]. Moreover, minimally invasive approaches such as minimally invasive TLIF or percutaneous pedicle screw fixation have gained popularity recently [8]. All lumbar spinal approaches require the use of proper instrumentation. The basic spinal fixation device consists of pedicle screws, connection rods, a cross-link device, and in some cases an interbody cage. Pedicle screws are placed into the vertebral bodies through the pedicles of vertebrae, the Harrington rods connect screws of adjacent vertebrae, and the cage is inserted into the intervertebral space (Figure 1). Such an interbody device enables distraction of disc space and successfully stabilizes the pathological segment. 

Over the last decade, pedicle fixation systems have evolved and modifications in spinal fusion techniques have been developed to increase fusion rates and improve clinical outcomes after LIF [3,7]. The new spinal fusion systems can vary in many strands including pedicle screw design (monoaxial or polyaxial), the method for the attachment to the spine, biomaterials used, and the screw–rod system connection (side-loading or top-loading) [7,9]. 

Spinal instrumentation efficiently improves interbody fusion rates and increases initial spinal stability. However, long-term increased stiffness of the stabilized segment provided by implanted screws and rods may lead to the development of adjacent segment degeneration (ASD) [10]. A possible pathogenic mechanism underlying that disease is the redistribution of stress at the adjacent levels, which results in increased intradiscal pressure and extended mobility in the neighboring segments [10,11]. These factors accelerate the degeneration of the adjacent segment intervertebral disc. ASD has been described mainly in the case of PLIF, but may also occur after other spinal fusion procedures. Recently, it has been noticed that ASD less frequently occurs after polyaxial pedicle screw fixation compared with monoaxial fixation [12].

The use of high-speed drilling, which is in some cases used during the LIF procedure, generates a lot of heat, which may cause thermal necrosis around the implant. To prevent this problem some authors showed that the use of CO_2_ as a coolant may be beneficial [13,14].

Regarding materials used for screw and rod manufacturing, metals, especially titanium alloys, are the most popular resources. Nowadays, many of them are made from titanium (mainly Ti-6Al-4V alloy). In the case of pedicle screws, that biomaterial can be also doped with hydroxyapatite, CaP, ECM, or tantalum. Regarding other materials used for rod fabrication, they include cobalt–chromium alloys and nitinol (nickel–titanium alloy) [9]. Furthermore, in the past few decades stainless steel (SS) has been commonly used in spinal instrumentation systems. Nowadays, SS is less often chosen as a biomaterial [10].

The purpose of this literature review is to summarize the physical and mechanical properties of metal alloys used for spinal instrumentation in LIF, which include fatigue strength, Young’s modulus, and corrosion resistance. Moreover, we also focused on describing biological response after their implantation into the human body.

Our article is mainly focused on titanium, cobalt–chromium, nickel–titanium (nitinol), and stainless steel alloys.

## 2. Physical and Mechanical Properties of Implant Important in LIF

The ideal biomaterial used in LIF should have high tensile and fatigue strength, Young’s modulus similar to that of the bone, and should be 100% resistant to corrosion to avoid mechanical failures. Therefore, the composition of the spinal rods and screws constitutes a crucial factor in defining the general functionality of the spinal instrumentation.

### 2.1. Fatigue Strength

One of the most important features of LIF implants is their fatigue strength. That property describes how long the spinal instrumentation can work without breaking down [15]. The cycling loading of the spine, which appears during daily activities, generates oscillating stresses on spinal instrumentation and may lead to a crack in the implant material. When the crack reaches a critical size, fatigue fracture of the material occurs, leading to the failure of the implant [15,16]. Remarkably, long cracks cause implant collapse slower than very small cracks, which are relative to the dimensions of the material micro-architecture [17]. Biomechanical performance and fatigue strength of spinal instrumentation significantly depend on the microstructure of the metal alloy of which it is made [18,19,20]. To increase the fatigue life of an alloy, many heat treatment techniques are implemented [21]. They include plasma-assisted microwave chemical vapor deposition, plasma nitriding, plasma etching, and deposition of amorphous diamond-like carbon (a-DLC) layers inoculated with nitrogen and silicon. It has been shown that these methods have a substantial influence on the surface characteristics and microarchitecture of alloys [22]. Furthermore, heat treatment allows for arranging an optimized balance of material features such as machinability, ductility, and stability [21].

Fatigue fractures almost always occur at stress concentration sites such as notches or the discontinuity of the geometrical structure of the material [16,17,23,24]. On the spinal rod’s surface, notches may be generated during manual contouring, which can impair the mechanical properties of the rod, especially its fatigue strength [16,25,26]. This phenomenon is known as the “notch effect”. Before metal rods are fixed to the patient’s spine, they are contoured to obtain optimal sagittal alignment of the spine [16,27]. Traditionally, contouring can be done by the surgeon with the use of the French bender. Alternatively, the rods can be anatomically designed and bent automatically by a machine before the surgical procedure. A biomechanical study by Yamada et al. [28] has shown that pre-contoured rods had a remarkably higher fatigue strength and ultimate load than intraoperative manually contoured rods at the same load condition. Moreover, pre-contoured rods not only reduce the risk of rod fracture but may also reduce operation time and bleeding and decrease the risk of infection in comparison to the manually bent rods [28,29]. This results from the manual contouring of the rods that requires a significant amount of time to adjust the proper shape of the rod. Furthermore, tightening of the screw leaves surface defects on the rods and may contribute to the notch effect formation, which has been described by many authors [16,17,18]. Therefore, avoiding severe tightening of the screws is recommended.

Recently, some studies have shown the negative influence of direct electrocautery use on the mechanical features of metal alloys [30,31,32]. According to a biomechanical study by Zobel et al. [31], electrocautery contact with the material was found to significantly decrease the fatigue strength of the Ti-6Al-4V titanium alloy. Even after a short contact with the electrode, the fatigue strength reduced remarkably. When electrocautery contact is applied at high-stress concentrations areas of instrumentation, it can be a notable problem for the mechanical properties of the implant and in extreme cases, it may lead to implant breakage [31,32]. Moreover, this problem was reported by many case studies in hip replacement surgery [32,33,34]. However, it remains unclear whether a decrease in fatigue strength after electrocautery contact depends on the material and whether it is determined by the type of the implant [31]. Furthermore, there is a lack of reports describing that issue in the case of other metal alloys. Regardless of that, spine surgeons should pay special attention and avoid any contact of the active electrocautery electrode with implants during the revision surgery, especially in the case of titanium implants and areas of the implant with high-stress concentrations. 

### 2.2. Young’s Elastic Modulus

A further property, which is crucial for the metal alloy to be useful as the material for the spinal implant, is its elasticity, which is the ability of a metal to resist distorting influence and return to its initial shape. This feature of a material can be described by a physical quantity known as Young’s elastic modulus. The value of the elastic modulus of human cortical bone ranges from 10 to 30 GPa [35]. The perfect alloy for use in spinal fusion systems should have Young’s modulus as similar as possible to the bone. That prevents a phenomenon called a “stress shielding effect”. That term refers to the reduction in bone density around the implant due to bearing of the majority of the mechanical load by the instrumentation. Normally, the bone experience stresses and remodels in response to the loadings. Therefore, due to decreased load, bone atrophy progressively occurs and it may result in implant loosening and failure [36].

Contemporary material engineering enables the development of metallic biomaterials, which have a modulus of elasticity more similar to human bone. They include different compositions of metals in alloys, which impact their mechanical properties and also materials with various porosity. Creating pores in the alloys not only improves osteointegration of the implant due to the better ingrowth of bone tissue into them but can also affect the value of Young’s modulus of the material. When the porosity of alloy increases, strength and elastic modulus of alloy decrease linearly [37,38]. Therefore, designing and manufacturing implants with the value of the elastic modulus close to that of human bone is possible.

### 2.3. Corrosion Resistance

The perfect biomaterial should also be corrosion resistant in any environment, especially in the internal environment of the body over any period of time. Generally, corrosion is a progressive degradation of a material resulting from its interaction with the extracellular body environment [39]. That environment contains a lot of ions of sodium, calcium, potassium, magnesium, chloride, phosphate, and bicarbonate, which may be potentially very corrosive factors [15,39].

In the literature, there are three different types of corrosion—fretting corrosion, crevice corrosion, and galvanic corrosion. Each of them has been observed in metallic spinal fusion systems [40,41]. Fretting corrosion develops as a result of mechanical damage from repeated micromotion and friction over time, occurring during the patient’s daily activity. It leads to the release of debris into the surrounding tissue [40]. This type of corrosion is determined by multiple factors such as the design of spinal instrumentation, used metal alloy, electrochemical environment, and load conditions [42]. Crevice corrosion results from exposing the metal to a surrounding tissue fluid, which can induce a local corrosion process by the point destruction of the passive oxide film [43]. Galvanic corrosion is the result of the presence of two different metals in contact with each other in the fluid environment [40]. The use of dissimilar metal alloys in the same spinal instrumentation systems could improve its mechanical features. On the other hand, mixing dissimilar metals in spinal implants brings with it an increased risk of inducing galvanic corrosion [40,42,44]. However, biomechanical studies conducted in 0.9% sodium chloride at 37 °C and retrieval analyses of spinal instrumentation have shown no evidence of galvanic corrosion in spinal constructs made of different metals [42,45,46]. After the literature review, we compared breakdown potential to assess corrosion resistance of each discussed metal alloy (Table 1). Materials with breakdown potential below 300 mV are regarded as unacceptable. The value of breakdown potential above 600 mV is considered corrosion resistance. Materials with marginal breakdown potential which ranges from 300 mV to 600 mV should be tested under the indicated use [47].

Most metallic alloys used in LIF are passive metals, which means that they have a stable oxide film on their surface [48]. That layer plays an important role in corrosion protection and the loss of its stability results in inducing the corrosion. In the presence of the above-mentioned ions in the surrounding environment, especially chloride ions, the passive film may be damaged [49]. Mean chloride ion concentration in interstitial body fluids is 113 mEq/L, which can induce corrosion in metallic implants [50]. Moreover, cycling loading, micromotion resulting from fretting, and other mechanical factors may also discontinue the passive layer on the surface of the implant [51].

Corrosion has a negative impact and not only leads to failure of the implant but also may leach debris and metal ions that could be harmful to the surrounding tissue. Moreover, some studies of spinal implants have detected elevated serum metal ion levels [43,52,53]. Other studies have found metal debris in lymph and organs such as the liver, spleen, and kidneys [54,55]. Metal ion release induces biological complications such as toxicity, hypersensitivity, and also cancerogenicity [39]. This phenomenon has been correlated with the output of cytokines and metallic proteases by activated macrophages, neutrophils, and T lymphocytes [56]. Other noted complications include pseudotumor and particle-induced osteolysis [57,58]. Localized neurological damage associated with rod breakdown has also been noted in several case reports [59,60,61].

**Table 1:** Quantitative comparison of mechanical properties of titanium alloys, cobalt–chromium, nitinol alloys, and stainless steel 316 L.

Alloy	Ultimate Tensile Strength [MPa]	Yield Strength [MPa]	Fatigue Strength [MPa]	Young Modulus [GPa]	Corrosion Resistance (Breakdown Potential) [mV]	References
Commercial Pure Titanium (CP-Ti)	240–550	170–480	430	115	9000	[21,62]
Ti-6Al-4V	930	860	500	110	25,000	[21,62,63]
Ti-24Nb-4Zr-8Sn (Ti2448)	665 ± 18	563 ± 38	375–500	53 ± 1	nd	[21,64,65]
Cobalt–Chromium	655	450	310	210	870	[30,62,63,66]
Nickel–Titanium	895	195–690 (austenitic phase) 70–140 (martensitic phase)	nd	40–75	>1000	[63,65]
Nickel–Titanium (CS 64% porous)	nd	~700	nd	1	772	[67]
316L Stainless Steel	490–1350	190–690	146	210	400–600	[21,47,62,67,68]

## 3. Mechanical Characteristics of the Most Frequently Used Metal Alloys in LIF

### 3.1. Titanium

Among all the metallic alloys used in the manufacturing of spinal instrumentation for LIF, titanium alloys are the most common materials. They owe their popularity to their excellent biocompatibility, superior mechanical properties, great corrosion resistance, and appropriately low Young’s modulus and generate minimal artefacts on computed tomography or magnetic resonance imaging [17,37,69,70]. These properties are highly preferable for biomedical applications. Due to the low elastic moduli and quite often observed notch effect of the titanium rods, titanium alloys are more often used in spinal screw fabrication than the spinal rods [9,16]. In contact with the air, a passive oxide film (TiO_2_) forms on the surface of the titanium. This layer is probably responsible for resistance to corrosion, chemical inertness, and stability of that metal [37]. 

There exist two well-known allotrophic phases of titanium—α and β phases. The type of alloy depends on the allotrophic phase of titanium which has been applied. Thus, we distinguish between α, near-α, α–β, and β alloys of titanium (Table 2).

Ti-6Al-4V alloy (α–β type alloy) is the most frequently used titanium alloy for spinal fixation devices [48,72,76]. Despite biocompatibility, excellent corrosion, and mechanical resistance of that alloy, its elastic modulus (~110 GPa), which is higher in comparison to human bone, may induce a stress-shielding effect and result in pedicle screw loosening and bone absorption [15,76,77,78]. Moreover, some studies have shown that Ti-6Al-4V accelerates the development of adjacent segment disease [78]. However, compared with other non-titanium metallic alloys used in spine fusion systems, Ti-6Al-4V has a relatively low modulus of elasticity and the stress-shielding effect is not as strong. Coating the pedicle screws with various materials such as PMMA, hydroxyapatite, extracellular matrix, and titanium plasma spray in tantalum was developed to improve the fixation and pull-out strength of the Ti-6Al-4V screws [79]. Many studies have successfully shown that coated screws may significantly increase resistance against pull-out force in comparison to uncoated screws [80,81,82]. Regarding potential toxicity associated with the leaching of the vanadium and aluminum ions from the Ti-6Al-4V implants, the amount of these metals released is minimal and does not induce suspected health problems, such as neurological or enzymatic disorders [83]. 

To better adjust the elastic modulus of titanium biomaterials to the cortical bone, increasing the β phase percentage in the alloy is an effective way [69]. One of them, in which this method was applied, is Ti-24Nb-4Zr-8Sn (Ti2448 alloy, α–β type). This material, drawing the attention of many researchers, has a lower Young’s modulus (~49 GPa) than Ti-6Al-4V and shows no toxic features [84]. Therefore, due to the elastic modulus value being more similar to human bone, the stress-shielding effect may be significantly less observed. It has been confirmed in a study conducted by Qu et al. [85] on a porcine model, which compared stress-shielding effects between Ti-24Nb-4Zr-8Sn alloy and Ti-6Al-4V alloy. Another low-modulus titanium alloy is the Ti-45Nb alloy (β type alloy) [73,74,75]. Besides the decreased value of Young’s elastic modulus, Ti-45Nb presents beneficial osteogenic features, which result from a high content of niobium [86]. Additionally, titanium alloys with increased content of β phase show increased corrosion resistance [87]. On the other hand, titanium alloys with low elastic modulus, such as β type alloys, usually also have low mechanical strength [78]. One of the well-known effective methods to increase the mechanical resistance of metals is precipitation hardening. However, due to irreversible changes in the crystalline structure, Young’s modulus increases, and corrosion resistance reduces due to this technique [88]. On the other hand, many studies have shown that severe plastic deformation (SPD) techniques, which include high-pressure torsion (HPT) [74,89,90,91], hydrostatic extrusion (HE) [92], and rolling and folding (R&F) [75], may improve the strength of β type titanium alloys without changing Young’s modulus. SPD techniques also improve corrosion resistance through the thickening of passive film [91]. However, alloys containing niobium, molybdenum, wolfram, or tantalum are expensive to manufacture due to the rarity and high melting points of these metals [48]. Thus, β type alloys such as Ti-24Nb-4Zr-8Sn and Ti-45Nb, despite their appropriately low Young’s modulus, excellent corrosion resistance, and sufficient mechanical properties after SPD processing, may have problems with spread of their use in LIF devices due to the very high costs of production.

Decreasing Young’s modulus of titanium alloys may be also achieved by creating pores in them. A biomechanical study by Skolakova et al. [83] has shown that Ti alloy with the addition of 30 wt.% pore-forming agent (PA) obtained with self-propagating high-temperature synthesis (SHS) exhibits a very similar elastic modulus (~9 GPa) to human bone with good corrosion resistance. However, mechanical strength decreased after the SHS procedure and further studies are necessary to evaluate the usefulness of Ti with 30 wt.% PA in LIF.

### 3.2. Cobalt–Chromium

Another metal alloy which may be used in LIF systems is cobalt–chromium (CoCr) alloy. This biomaterial usually consists of 63% cobalt, 28% chromium, 5% molybdenum, and minor amounts of other metals [48]. Well-known applications of CoCr alloy as biomaterial include hip and knee joint implants, as well as crowns and implant abutments in dentistry [48,83,93]. CoCr is characterized by higher fatigue life and strength, increased stiffness, and better resistance to notch effects in comparison to titanium alloys [18,23,94,95,96,97]. Due to its higher Young’s modulus (~210 GPa) [30] than titanium, CoCr spinal rods more effectively stabilize the spine and correct abnormalities of spinal curvatures such as scoliosis [96,98,99]. In the study by Willson et al. [100], CoCr rods demonstrated the least amount of shape loss in a radius of curvature compared with commercially pure titanium rods throughout the study.

However, high stiffness of CoCr rods may result in acceleration development of adjacent segment disease [99,101]. According to a comparative study by Han et al. [102], breakages of CoCr rods have been less observed than for titanium rods, but in the case of CoCr, they observed a more frequent occurrence of proximal junctional kyphosis (PJK), which is a form of adjacent segment degeneration. Moreover, a Young’s modulus significantly higher than that of human bone disqualifies CoCr alloy as a biomaterial for screw manufacturing due to the increased risk of stress-shielding effects. Furthermore, compared with titanium alloys, CoCr has lower corrosion resistance, which results in higher overall metal ion release from CoCr implants. Leaching cobalt ions from the alloy due to fatigue and biocorrosion may cause metallosis, neurological-related symptoms (such as deafness and blindness), hypothyroidism, cardiological and hematological problems, and also cancers [103,104,105,106]. To prevent these issues, coating of CoCr implants with ceramics such as calcium phosphate can decrease cobalt ion release and improve biocompatibility, which has been proven in a study by Bandyopadhyay et al. [107] with the use of the surface melting (LSM) technique for tribofilm formation.

### 3.3. Nitinol

Nickel–titanium (nitinol) is a metal alloy which consists of titanium and nickel in equal atomic percentages [48]. Among all the alloys used in spinal fusion devices, nitinol is characterized by a unique feature which is its superelasticity [48,83,108]. This phenomenon enables the nitinol implant to immediately return to an undeformed shape after removal of external load, even after large deformations. In this way, the use of this super-elastic alloy in LIF systems as rod material makes stabilization more dynamic and may prevent ASD occurrence [63]. Moreover, nitinol is used clinically in intravascular stents, osteosynthesis staples, and orthodontic wires [63,67,83]. 

Additionally, nitinol has Young’s modulus ranging from 40 to 75 GPa, which is optimal for biomedical applications. Moreover, nitinol fabrication by combustion synthesis (CS) enables tailoring its elastic modulus to that of human bone with great accuracy. After that procedure, metal alloy achieves high compressive strength with appropriately low Young’s modulus and excellent corrosion resistance [67]. According to Aihara et al. [67], general porosity of nitinol to obtain the best elasticity was found to be 64%.

Due to the formation of a passive titanium oxide film (TiO_2_) on the surface of the nitinol, it is considered a long-term corrosion-resistant and biocompatible alloy [63]. Therefore, coupling nitinol rods with titanium pedicle screws may be considered the best combination for spinal fusion devices due to its high resistance to galvanic corrosion [109]. The corrosion resistance of nitinol alloy is better than CoCr and 316L stainless steel, but inferior to that of Ti-6Al-4V [48,63]. However, the in vitro study combined with retrieval analysis of the nitinol, CoCr, and Ti-6Al-4V rods by Lukina et al. [63] has also shown that nitinol fretting corrosion patterns were worse compared with CoCr. That result may be an effect of lower resistance to fretting corrosion of the nitinol due to higher mobility of the rod. Moreover, intensive fretting may damage the passive oxide layer, whose restoration is relatively low. Thus, it may induce galvanic corrosion and deteriorate its overall corrosion resistance. As result, it affects the fatigue strength of nitinol and may release nickel ions into the blood. However, some studies have shown that the nickel ion levels in blood and tissues were not higher compared with the control group. In any case, to prevent fretting corrosion, coating nitinol rods with protective layers and enhancing the locking mechanism of the pedicle screws would be beneficial solutions [63].

### 3.4. Stainless Steel

Before introducing titanium alloy as a biomaterial for spinal construct manufacturing, stainless steel (SS) was the most popular metal alloy in this field. It is widely used for other biomedical applications such as bone fixation, cardiovascular systems, catheters, surgical instruments, or dental crowns. Surgical 316 L SS is the most common form of stainless steel for biomedical uses. This specific composition consists of 0.02% carbon, 10–14% nickel, 16–18% chromium, 2% manganese, 2–3% molybdenum, with the rest being iron. The high mechanical properties of this alloy are great advantages for use in spinal fixation. However, SS exhibits a significantly higher elastic modulus (210 GPa) [67] in comparison to human bone. Thus, the stress-shielding effect is strongly observed after SS implant application [110]. Regarding corrosion resistance, many studies have shown that it is significantly inferior compared with CoCr and titanium alloys [40,42,45]. Long-term biomechanical tests by Singh et al. [45] have shown that both CoCr and titanium constructs were more resistant to the fretting corrosion compared with SS. Moreover, during the corrosion process, SS constructs have produced a noticeably greater volume of debris than titanium or CoCr instrumentation systems [45]. Therefore, stainless steel should no longer be in used in spinal surgery.

## 4. Biological Response to Metal Implants Used in LIF

Implanted material elicits a biological response driven by immune cells at the site of insertion as well as systematically [111]. These reactions are subdivided into innate (primary cellular response with no previous exposure) and adaptive (a specific type of reaction induced after earlier exposure to the antigen) and are responsible for wound healing, fusion, and also adverse reactions, i.e., hypersensitivity [112,113,114]. A comprehensive understanding of cellular and molecular pathways is essential to identify preferable characteristics of implanted biomaterial to obtain fusion and avoid implant loosening.

### 4.1. Wound Healing

Bone decortication and bleeding are caused by the inserted implant triggering intramembranous ossification, a process essential for successful bone remodeling and incorporation, leading to arthrodesis [115,116,117]. Implantation of the metal screw is followed by adsorption of a proteinaceous layer [118] (made of serum molecules, water, and proteins) on the implant surface followed by the formation of a blood clot that recruits inflammatory cells on the side of the instalment and initiates provisional matrix formation [119]. Complex molecular processes following wound healing and interbody fusion are characterized by three main phases described initially by Boden et al.: inflammatory, reparative, and remodeling phases [120]. The acute inflammatory phase lasts up to three weeks and is defined by the migration of inflammatory cells including lymphocytes, leukocytes, and macrophages and secretion of cytokines at a site of merging [121]. Pro-inflammatory IL-6 and C reactive protein are the key components of this stage. Here, it is vital to distinguish the healing process from complications such as surgical site infection [122]. According to Thalander and Larsson, the levels of CRP and IL-6 reach their peak on post-operative day 3 and then decrease with time [123]. If otherwise, the patient should be suspected as having infectious process development [124]. The prolonged, unresolved inflammatory response may lead to chronic inflammation, the development of granulation tissue, and the formation of a fibrous capsule—a host of reactions that lead to implant dysfunction. The main process following the second, reparative stage is the differentiation of progenitor cells, neovascularization, and resorption of necrotic tissue [125]. The fusion mass subsequently matures at the entry point (transverse processes in the case of lumbar fusion) which is followed by the migration of the ossification process to the central zone [126]. Consequently, the last, remodeling phase occurs, which is defined by further maturation of new bone tissue and an increase in cortical to cancellous bone ratio [127]. The evolution of these processes is regulated by the expression of various genes responsible for the translation of bone morphogenic proteins—BMPs 2, 4, and 6 [128]. 

The success and intensification of the mentioned processes are largely dependent on the properties of the implanted material. In recent years, instead of producing materials diminishing host responses, designed implants are made of biomaterials that aim to modulate immunologic reactions towards enhanced fusion [129]. Desirable attributes which define biocompatibility leading to osteointegration include osteogenicity, the capacity to provide stem cells and osteoblast enabling new bone formation [130], osteoinductivity, recruitment of osteogenic growth factors [131], and osteoconductivity, ensuring appropriate conditions for the ingrowth of bone-forming elements and providing a scaffold for osteogenic cells and neovascularization [132,133]. 

The opposite, undesirable chronic inflammatory reaction on the implant/bone interface caused by metal debris or ions results in peri-implant bone osteolysis (PPOL) [134], a process that threatens permanent implant endurance. 

### 4.2. Foreign Body Reaction

Acceptance of implanted instrumentation is dependent on various innate reactions that are collectively described as foreign body response (FBR) reactions that begin immediately after insertion [135]. Injury to the bone tissue results in the recruitment of immune cells and activates coagulation and complement pathways [136]. Subsequently, extravasated proteins, i.e., fibronectin, fibrinogen, and vitronectin, are adsorbed on the metal–tissue interface, which contributes to the formation of a provisional matrix in the vicinity of the implanted material and, after migration of macrophages, contributes to the formation of foreign body giant cells [137]. Due to potency variation of the Vroman effect [138], a process of competitive protein adsorption and desorption on a metal surface, foreign body response differs among materials used in fusion [139]. This event is followed by neutrophil recruitment which further enhances the inflammatory process, i.e., activation of mast cells and attraction of monocytes and, consequently, the transformation of monocytes to macrophages [140]. Accumulation of cells and proteins on biomaterial through integrins, particularly aMB2 [141,142], creates a privileged microenvironment between the implant and host tissues. Due to the process called “frustrated phagocytosis”, macrophages release degradation enzymes and ROI that aim to break down implanted biomaterial [135]. Unsuccessful degradation leads to the transition from an acute to chronic phase hallmarked by a switch from M1 to M2 macrophage polymerization [143]. Whereas M1 macrophages, referred to as classically activated, are pro-inflammatory, M2 macrophages, which are alternatively activated, are responsible for anti-inflammatory reactions that participate in wound healing. M1 macrophages produce TNF1, IL-1, and IL-6 and are linked to the Th1 type of immune response [144]. M2 macrophages produce anti-inflammatory cytokines including IL-4, IL-10, and IL-13 and are associated with Th2 immune response [145,146]. Although, naturally, the preponderance of M2 polymerized macrophages heralds a typical wound healing process, in the case of biomaterial implantation their predominance marks a shift from elimination to the tissue healing process. In the chronic phase, macrophages fuse to create foreign body giant cells on the implant’s surface [147]. This process is followed by neovascularization mediated by VEGF and PDGF and terminates when the created capsule becomes entirely isolated from neighboring tissues [148].

### 4.3. Response to Implant Wear Debris and Metal Ions

Biological reactivity to metal implant debris is the main factor that determinates successful spinal implant fusion and is the leading cause of undesirable implant rejection. There are two main types of immunologic reactions induced by implanted metals: innate and adaptive [149,150].

Resident macrophages are responsible for the slow elimination of metal wear debris particles due to subtle innate, non-antigen-specific immune responses [151]. As a result, no immunologic memory is preserved after exposure. Apart from that, their activation inaugurates a response to metal ions in a hypersensitivity reaction, a delayed hypersensitivity response (DTH), which is a type of adaptive T lymphocyte and antigen-dependent reaction resulting in immunologic memory development. Both processes cause pain, implant loosening, and, in consequence, aseptic implant failure [152]. 

### 4.4. Innate Reaction

Innate reactions directed to metal implant wear debris are central immune responses that lead to implant failure. Identification and uptake of wear particles activate macrophage pattern recognition receptors (PRRs) and initiate the release of pro-inflammatory cytokines (IL-1, IL-6, TNFα, PGE-2), chemokines (monocyte chemoattractant protein (MCP-1), macrophage inflammatory protein (MIP-1a)) and pro-osteoclastic factors (receptor activator of nuclear factor kappa B ligand (RANKL)) [153,154]. PPRs recognize stimuli composing pathogen- or danger/damage-associated molecular patterns (PAMPs and DAMPs) and are divided into Toll-like (TLRs) and C-type leptin receptors (CLRs) [155]. Metal particles directly activate TLRs resulting in activation of NLRP3 inflammasome and promotion of interleukins such as IL-1B secretion [156] and recruitment of myeloid-lineage cells [157,158]. Inflammasomes function as the main regulator of the wound healing process and cause the production of various immune mediators including IL-1B, IL-6, CXCL8/L8, CCL/MCP-1, TNFα, nitric oxide, etc. [159,160].

Pro-inflammatory cytokines, i.e., IL-1B and TNFα, enhance the expression of RANKL and inhibit the expression of suppressors of osteoclastogenesis (i.e., osteoprotegerin), impede mesenchymal stem cell differentiation into osteoblasts, and even cause osteoblast apoptosis [161,162]. RANKL binds to RANK receptors on osteoclast precursors (OCPs) and activates nuclear factor kappa light chain enhancer of activated B cells (NF-κB) and mitogen activated protein kinase (MAPK) signaling pathways, resulting in augmentation of bone resorption and consequent implant failure [163,164].

### 4.5. Adaptive Response

Adaptive immunity depends on the activity of lymphocytes, is antigen dependent, and results in the formation of immunologic memory following exposure. Metal particles, i.e., ions, act as haptens with high immunogenic potential. Some, especially when present at excessive levels, are able to initiate an adaptive immune response, in the form of antigen-dependent metal allergy or type IV delayed type hypersensitivity (DTH) [165]. Due to the preponderance of leukocytes among macrophages, giant cells, and other cells within peri-implant pseudotumor/granule tissues, it has been proposed that adaptive reactions play an important role in metal implant failure. These reactions are characterized by vasculitis and infiltration of the vessel wall, perivascular space, endothelium edema, and necrosis.

Among known metals, beryllium [166], chromium [167], cobalt [168], nickel [169], tantalum [170], titanium [171], and vanadium [172] belong to metals considered as sensitizers. While nickel is known as the most common allergen in humans, chromium and cobalt are frequent hypersensitivity inducers [173]. The available literature provides data that demonstrate relevant dependence between the amount and size of metal debris and initiation of hypersensitivity reactions [152,174]. In an event of suspicion of metal allergy, a lymphocyte transformation test is advised.

### 4.6. Biocompatibility of the Most Frequently Used Metal Alloys in LIF

#### 4.6.1. Titanium

Out of all available alloys, commercially pure titanium possesses the distinctive feature of osteointegration, an ability to create a direct structural and functional connection between the implant and bone tissue without the production of any soft tissues in between. Following implantation, on a micro- and nanometer scale, titanium is covered by a thin Ti oxide layer, proteinaceous layer, a slender cell layer, calcified region, and bone tissue [175]. The efficacy and rate of the osteointegration process are enhanced by modification of Ti properties by altering surface micro- and nanostructure, roughness, hydrophilicity, biological surface treatment, or chemical addition [176,177,178,179,180]. Titanium presents early macrophage polarization into M2 macrophages, resulting in early anti-inflammatory/reparative (ARG1, CD4+) response [181]. Moreover, the change in surface structure modulates the degree of polarization into pro-healing M2 macrophages [179]. Through that mechanism, ceramic coatings with the use of hydroxyapatite enhance osteogenic bone response [182]. In a study conducted by Trinidade et al., during the first 10 days following titanium implantation, bone suppression markers were downregulated in an in vivo rabbit model [183]. In a further study on an animal model that investigated the osteointegration process in the first 4 weeks following implantation, the genetic evaluation revealed suppression of bone resorptive genes including ANKL, OPG, TRAP, and CathK compared to a sham control group [183,184].

#### 4.6.2. Titanium Alloys

Commercially pure titanium (CP-Ti) and titanium alloy Ti-6Al-4V are both widely utilized in the operative field as metal implants. Although there are growing concerns about toxicity of vanadium [185], in a study conducted by Doe et al., despite achieving highest concentrations after 4 weeks of implantation, toxic levels have not been reached in animal models [186]. The search for alternatives has driven adaptation of other alloys such as Ti-6Al-7Nb, Ti-5Al-2.5Fe, Ti-15Mo, Ti-13Nb-13Zr, Ti-12Mo-6Zr-2Fe, Ti-35Nb-5Ta-7Z, and Ti-29Nb-13Ta-4.6Zr. Some components present great biocompatibility (i.e., Au, Ca, Mg, Mo, Nb, Pt, Pd, P, Sr, Sn, Si, Ta, Ti, and Zr) but others, i.e., Al, Ag, Be, Cr, Co, Cu, Cr, Fe, Mn, Ni, V, and Zn, display toxic reactions both in in vitro and in vivo studies [187,188,189]. Hence, alloys comprising biocompatible elements—Ti-39Nb-6Zr (TNZ) and Ti-39Nb-6Zr + 0.45Al (TNZA)—started to gain growing attention [190]. Nevertheless, some studies suggest a detrimental effect of Al and its involvement in neurodegenerative disorders or metabolic diseases [104,191] which might necessitate additional research that would evaluate its employment in spinal surgery.

#### 4.6.3. Cobalt–Chromium

As reported in recent studies, metal ions and wear particles have the potential to leach from metallic implants, eliciting adverse immunologic reactions [192,193,194]. In in vitro studies conducted by Moeed Akbar et al., Cr (6+) and Co (2+) ions affected primary human lymphocytes by inducing apoptosis, inhibiting T lymphocytes and impeding the release of IL-2 through yet unknown mechanisms [195]. This is also viable with low circulating ion levels [196]. Cobalt and chromium alloy particles as well as their ions activate the NLR family pyrin domain containing 3 (NLPR3) inflammasome and caspase-1 mediated pathway, leading to activation of pro-interleukin IL-1B and pro-IL-18 as a part of an innate immunologic response [197,198,199]. This in turn activates NFκB that stimulates various pro-inflammatory responses [156]. 

#### 4.6.4. Nitinol

Nickel is the most common contact allergen and affects up to 10% of representatives of the Caucasian population [200,201].

Nitinol has similar biocompatibility to titanium and it is better than that of stainless steel, therefore it shows promising potential in clinical application. It has similar fibroblast and osteoblast proliferation potential to Ti and SS [202]. In a study conducted by Haider et al., Ni ions exhibited greater toxicity on HUVECs than Cr and Ta [203]. Biocompatibility of nitinol then depends on Ni which is advised not to exceed 50% [204]. Its clinical use is limited by Ni toxicity, which can cause inflammation, DNA damage, ROI formation, etc. Although the level of tolerance is not established yet, due to a lack of evidence in in vitro studies, even low concentrations are proved to limit proliferation in in vitro experiments [185].

Studies have shown that due to the passive titanium oxide layer coating the surface of the nitinol implant, the likelihood of releasing the ions into the recipient’s tissues is similar to that of stainless steel and cobalt-based alloys. Instances of Ni ions being released have been noted as more frequent in parts of the implant covered with a thicker oxide layer, and they are more prone to breaking [205]. In a study performed by Nagaraja et al., on healthy lab minipigs with implanted optimally surfaced nitinol stents, no adverse effects of nitinol on kidneys or the hematopoietic system were observed. Nevertheless, non-optimally surfaced stents caused vessel stenosis and inflammation [206]. This outcome is coherent with other studies and proves that avoidance of wear debris and adverse effect of nitinol is feasible by surface processing, i.e., DLC coating [207,208]. 

#### 4.6.5. Stainless Steel

The most commonly used types of stainless steel implants are ones made from the SAE 316L alloy. This specific composition consists of 0.02% carbon, 10–14% nickel, 16–18% chromium, 2% manganese, 2–3% molybdenum, with the rest being iron and it shall be treated as a whole, as well as the sum of its’ metals and as such. All of the possible reactions of the aforementioned metals shall be taken into consideration, as they have been reported to dissociate into the recipient’s tissue [209,210]. Cytotoxicity of these metal particles as well as immediate hypersensitivity caused by them have been characterized by peri-implant infiltration of macrophages and lymphocytes with CD68+, CD14+, and HLA-DR+ macrophages, as well as formation of CD3+ T cell and CD20+ B cell congregates [209,211].

Other than direct primal inflammation, stainless steel implants have been shown to cause apoptosis and chronic inflammation dependent on CD8+ cells [212]. As for metal particles, histological research has shown a high expression of HLA-DR active cells proximal to the SS area. T cell lymphocytes have been detected up to 6 months after initial surgery, suggesting chronic inflammation; it is worth noting that this behavior is consistent with both SS and titanium implants [212]. There have been reported cases of type IV delayed allergic reaction to implanted stainless steel plates. It is mediated by antigen-presenting cells and T lymphocytes causing the buildup of lymphocytes, histiocytes, and foreign body giant cells as well as inflammation of the implant area [213,214].

As for specific components of the alloy, iron exhibits an important role in modulating immune response from lymphocytes, NK cells, T cells, monocytes, and macrophages. Research done on mice has shown that iron oxide, depending on doses and particle size, either suppresses or enhances immune responsiveness. There are studies suggesting that the surface texture and roughness also play a role in the severity of the reaction, as macrophages tend to adhere more consistently to grooved surfaces of the metal and, in turn, induce a more significant inflammation [215].

Stainless steel implants that undergo corrosion have also been found to release hexavalent chromium into the recipients’ tissues and bloodstreams [216]. There is a small concentration of manganese ions that could be released from the implant, and there have been cases of type IV allergic reaction to this component [217]. However, some research has shown that stimulation of anti-viral immune responses is achievable by implementing manganese ions. They have been reported to stimulate M1 macrophages and CD8+ T cells as well as boost the host’s adaptive immunity [218]. Molybdenum has been reported to induce an inflammatory response by activating the NLRP3 inflammasome in macrophages by stimulating the secretion of IL-1β, which is hypothesized to be one of the reasons behind peri-implant tissue inflammation [219]. 

The biocompatible characteristics of above-mentioned metal alloys have been compared in Table 3. 

## 5. Summary

Both mechanical features of metal alloy and the biological response induced by the metal implant are essential for the efficiency of LIF. An appropriate fatigue strength, mainly determined by the microstructure of the metal alloy, decreases the risk of implant fractures. Corrosion resistance has an influence not only on implants’ mechanical performance but also can prevent releasing metal ions into the surrounding tissue and bloodstream. Furthermore, decreasing the Young’s modulus may avoid the “stress-shielding” effect. On the other hand, implanted material elicits a biological response driven by immune cells at the site of insertion. That response is determined by wound healing, foreign body reaction, response to implant wear debris and metal ions, innate reactions, and adaptive immunity. All these properties are crucial to avoiding implant failure and obtaining spinal fusion.

Every metal alloy discussed in this paper has its advantages and disadvantages. However, high Young’s elastic modulus, poor corrosion resistance, and allergic and inflammation reactions disqualify stainless steel from use in LIF instrumentation. Cobalt–chromium alloy as a material eliciting adverse immunologic reactions and demonstrating high elastic modulus does not appear to be a good alternative. The use of superelastic and biocompatible nitinol may reduce the rate of adjacent segment disease. Unfortunately, its clinical use is limited by the toxicity of nickel. However, surface processing (e.g., DLC coating) may prevent this limitation and in the future enhance the popularity of this alloy in spinal instrumentation. Despite decreased, but still relatively high, elastic modulus, titanium alloys, especially the most popular Ti-6Al-4V alloy, remain a standard biomaterial for LIF instrumentation. Moreover, some manufacturing techniques (e.g., surface processing) can decrease Young’s modulus while preserving the mechanical and distinctive osteointegration properties of these alloys. On the other hand, the osteogenicity of titanium can be enhanced by ceramic coatings. Furthermore, adverse immunologic reactions are not frequent in comparison to other alloys. Therefore, titanium alloys currently represent the safest and the most effective materials among the discussed metal alloys for implants in LIF.

There are some more unknown aspects in designing implants and their material properties that can connect lumbar interbody fusion to other spine disorders such as cerebrospinal fluid leakage and CM-I that can be studied in future works [223]. In the future, further achievements in biomaterial engineering may help to obtain desirable biological and mechanical features of spinal implants to provide effective and safe spinal fusion.

## Figures and Tables

**Table 2:** Composition of titanium alloys used in lumbar interbody fusion.

Titanium Alloy	Chemical Composition (%wt)	Phase Type	References
Commercial pure titanium (CP-Ti)	99–99.5% Ti	α type	[71]
Ti-6Al-4V	6.29% Al	α–β type	[31]
	4.02% V		
	<0.2% other elements		
	Ti balanced		
Ti-24Nb-4Zr-8Sn	24% Nb	α–β type	[72]
	4% Zr		
	8% Sn		
	<0.3% other elements		
	Ti balanced		
Ti-45Nb	44.94% Ni	β type	[73,74,75]
	<0.5% other elements		
	Ti balanced		

**Table 3:** Comparison of biocompatible characteristics of titanium, cobalt–chromium, nitinol, and stainless steel alloys.

Alloys	Foreign Body Reaction	Innate Reaction	Adaptive Response	Healing Process	References
Titanium	Formation of foreign body giant cells is common	Prolonged presence of neutrophils	Osteointegration	Enhanced osteogenic response	[182,183]
CoCr	Fewer instances of foreign body giant cell formation than in SS	Induction of IL-1B and T cell lymphocyte proliferation	Decrease in cytokine production over time	Enhanced angiogenesis	[197,198,199,220]
Nitinol	Inflammatory response due to Ni ions being released	Inflammation in presence of macrophages and lymphocytes	Rare cases of type IV delayed hypersensitivity response	Osteointegration higher than titanium	[221,222]
SS	Higher inflammatory response than in other analyzed materials	Inflammation in presence of macrophages and lymphocyte congregates	Buildup of lymphocytes, histiocytes, giant cells and inflammation	Increased inflammatory response slows down the healing process	[209,211,213,214]

## Data Availability

Not applicable.
